# Clinical epidemiology and viral genomics insights from a Chikungunya fever outbreak in South China, 2025

**DOI:** 10.3389/fcimb.2026.1762631

**Published:** 2026-01-23

**Authors:** Fangfang He, Yufeng Liang, Yuanxin Gong, Peihan Li, Jiayin Yu, Chuhong Wei, Jian He, Fenxiang Li, Ruolan Yu, Wei Yang, Cuixiang Yi, Aiyang Lin, Wenting Yu, Peng Li, Jintao Li, Huacheng Yan

**Affiliations:** 1 Infection Control Department, Lecong Hospital of Shunde, Foshan, China; 2 Southern China Center for Disease Control and Prevention, Guangzhou, China; 3 Biosafety Department, School of Basic Medical Sciences, Army Medical University, Chongqing, China; 4 The Chinese PLA Center for Disease Control and Prevention, Beijing, China

**Keywords:** Chikungunya fever, China, clinical characteristics, phylogenetic analysis, risk factors

## Abstract

**Background:**

Chikungunya fever (CHIKF) is a mosquito-borne viral disease characterized by fever, rash, and severe joint pain. However, these classical descriptions are based overwhelmingly on the Indian Ocean and Caribbean lineages. With the recent introduction and spread of the Middle Africa lineage (MAL) into Asia, understanding its clinical presentation in new populations, such as Chinese, has become a public health priority. Whether the recently introduced MAL causes comparably severe disease in China remains unknown.

**Methods:**

We enrolled 415 laboratory-confirmed cases of Chikungunya virus (CHIKV) infection during an outbreak in Foshan, China. Clinical manifestations, laboratory parameters, and whole-genome sequencing data were integrated to quantify the symptom burden from three different perspectives using multivariate logistic regression, and to trace the viral source via maximum-likelihood phylogenetic analysis.

**Results:**

Compared with the classical phenotype, the MAL outbreak in China was appreciably milder. The most common clinical manifestations were arthralgia (83.61%), fever (74.46%), and rash (61.93%). Multivariate logistic regression showed that older age (OR = 0.979, P = 0.029) and male sex (OR = 0.528, P = 0.038) were negatively correlated with the occurrence of higher symptom burden, while prolonged fever (OR = 8.156, P < 0.001) was a significant risk factor. Reduced estimated glomerular filtration rate and thrombocytopenia were associated with longer disease duration. Phylogenetic analysis revealed that the outbreak-associated CHIKV strains belonged to MAL and harbored the E1-A226V and E2-I211T mutations.

**Conclusion:**

These findings provide an evidence base for clinical management and prognostic assessment during CHIKF outbreaks and underscore the importance of monitoring laboratory parameters alongside molecular surveillance.

## 1 Introduction

Chikungunya fever (CHIKF) is an arthropod-borne viral disease caused by the Chikungunya virus (CHIKV), a member of the Togaviridae family. It is primarily transmitted through the bite of infected *Aedes* mosquitoes (Silva and Dermody, 2017). According to the latest classification system, CHIKV strains worldwide are divided into nine distinct lineages: Asian urban lineage (AUL), AUL-America lineage (AUL-Am), South America lineage (SAL), Middle Africa lineage (MAL), Indian Ocean lineage (IOL), East Africa lineage (EAL), Africa and Asia lineage (AAL), Sister Taxa to ECSA (sECSA) and West Africa (WA) (Nadim Sharif et al, 2021).

The epidemiological profile of CHIKF exhibits striking similarities to that of other arboviral disease such as dengue fever and Zika virus infection, particularly in terms of transmission dynamics and geographic distribution (Côrtes et al, 2023; Weaver et al, 2018; Zong et al, 2023). This shared epidemiological pattern, coupled with the expanding global range of competent mosquito vectors, has established CHIKF as an emerging public health concern across tropical and subtropical regions worldwide (de Lima Cavalcanti et al, 2022; Khongwichit et al, 2021). As of June 2025, local transmission of CHIKF has been reported in 119 countries and regions globally, with most cases concentrated in the Americas, Asia, and Africa (Li et al, 2025; Wan et al, 2025). In China, the first locally transmitted CHIKV outbreak—linked to an imported case—was reported in Foshan City, Guangdong Province, on July 8, 2025 (Guangdong Provincial Center for Disease Control and Prevention, 2025). By September 20, a total of 13, 299 confirmed CHIKF cases had been documented in Guangdong Province, with 9, 958 cases (accounting for 74.88%) concentrated in Foshan, reflecting a notably concerning epidemiological scenario. Compounding these challenges is the absence of specific antiviral therapies and commercially available vaccines, highlighting the urgent need to better understand the clinical progression and risk factors of CHIKF—knowledge critical to informing effective outbreak control strategies (Powers, 2018).

To fully characterize this outbreak, we systematically collected and analyzed clinical-epidemiological data, including patients’ demographic characteristics, clinical manifestations, and laboratory findings. Concurrently, we performed molecular characterization of the locally outbreak-associated CHIKV strain via whole-genome sequencing and phylogenetic analysis. The objectives of this work were to identify the clinical features and risk factors of the outbreak, define the causative CHIKV genotype and key viral mutations, thereby tracing the outbreak’s origin and clarifying its transmission dynamics.

## 2 Method

### 2.1 Study population

A descriptive cross-sectional study was conducted with data between July and August 2025 from 415 patients diagnosed with CHIKF admitted to Lecong Hospital of Shunde, Foshan, China.

### 2.2 Case definition

Suspected cases of CHIKV were defined as the presence of fever, rash, and arthralgia by the clinician. Laboratory confirmation was performed by real-time RT-PCR. A total of 415 cases were confirmed as acute CHIKF cases based on the presence of CHIKV RNA in the serum samples.

### 2.3 Data collection

Patients’ sociodemographic information, self-reported comorbidities, and clinical symptoms were collected using case record forms (CRFs) and semistructured health questionnaire. Hematological reports were obtained through the hospital test system including blood test, liver function test, kidney function test and C-reactive protein (CRP) level.

### 2.4 Statistical analysis

The Kolmogorov-Smirnov test was used to assess the normality of the data. Continuous variables that were normally distributed were compared using t-tests or analysis of variance (ANOVA), while those that were not normally distributed were compared using the Mann-Whitney U test or Kruskal-Wallis test. Nominal categorical variables were compared using the chi-square test or Fisher's exact test, and ordinal categorical variables were compared using the Kruskal-Wallis test. To identify factors independently associated with binary outcomes, multivariate logistic regression analysis was used to screen for risk factors and protective factors, with model fitting performed using the forward stepwise regression method. Variables were added stepwise based on their significance until no more variables met the criteria for entry into the model (*P* < 0.05). The model outputs included the regression coefficients (b) for each independent variable, along with their standard errors, Z-values, H- values and P-values. The exponentiated regression coefficients (exp(B)) represented the odds ratios (OR), which were used to measure the impact of the independent variables on the dependent variable. For regression analysis, patients were categorized based on the number of cardinal symptoms (fever, arthralgia, rash). "Symptom burden" was defined as a binary outcome, with low burden (0–1 symptom) and high burden (2–3 symptoms). Patients were also categorized based on fever severity, with "fever severity" defined as a binary outcome: mild group (normal temperature and low-grade fever groups) and severe group (moderate and high fever groups). Additionally, patients were categorized based on nucleic acid conversion time, with this time defined as a binary outcome: rapid conversion group (≤ 6 days) and slow conversion group (> 6 days). Correlation analyses were conducted using Pearson's correlation (for normally distributed continuous variables) or Spearman's rank correlation (for non-parametric data). All statistical tests were two-sided, with *P* < 0.05 considered statistically significant. The analyses were performed using R software (version 4.3.2).

### 2.5 Whole genome sequencing and phylogenetic analysis

PrimalScheme version 3.2.2(9) was used to design whole-genome targeted amplification primers for CHIKV. Whole-genome sequencing was performed on 92 CHIKV-positive patient samples using the DNBSEQ-G99 sequencing platform (MGI Tech). The reference strain, Réunion Island 80652-1-1-1 (GenBank: PV700165.1), was used for read mapping with BWA version 0.7.18 and SAMtools version 1.20.0 (Danecek et al, 2021; Li and Durbin, 2010). Variant calling and genome assembly were conducted using iVar version 1.4.4 (Grubaugh et al, 2019). A global collection of 1, 990 complete genome sequences of CHIKV (available up to September 1, 2025) was downloaded from NCBI for phylogenetic analysis. Multiple sequence alignment was performed using MAFFT version 7.525 (Rozewicki et al, 2019). The phylogenetic tree was constructed with FastTree version 2.2.0 (Price et al, 2010) and visualized using the iTOL online server (Letunic and Bork, 2024).

## 3 Results

### 3.1 Epidemiological profile of CHIKF outbreak in Shunde, Foshan

**Figure 1:**
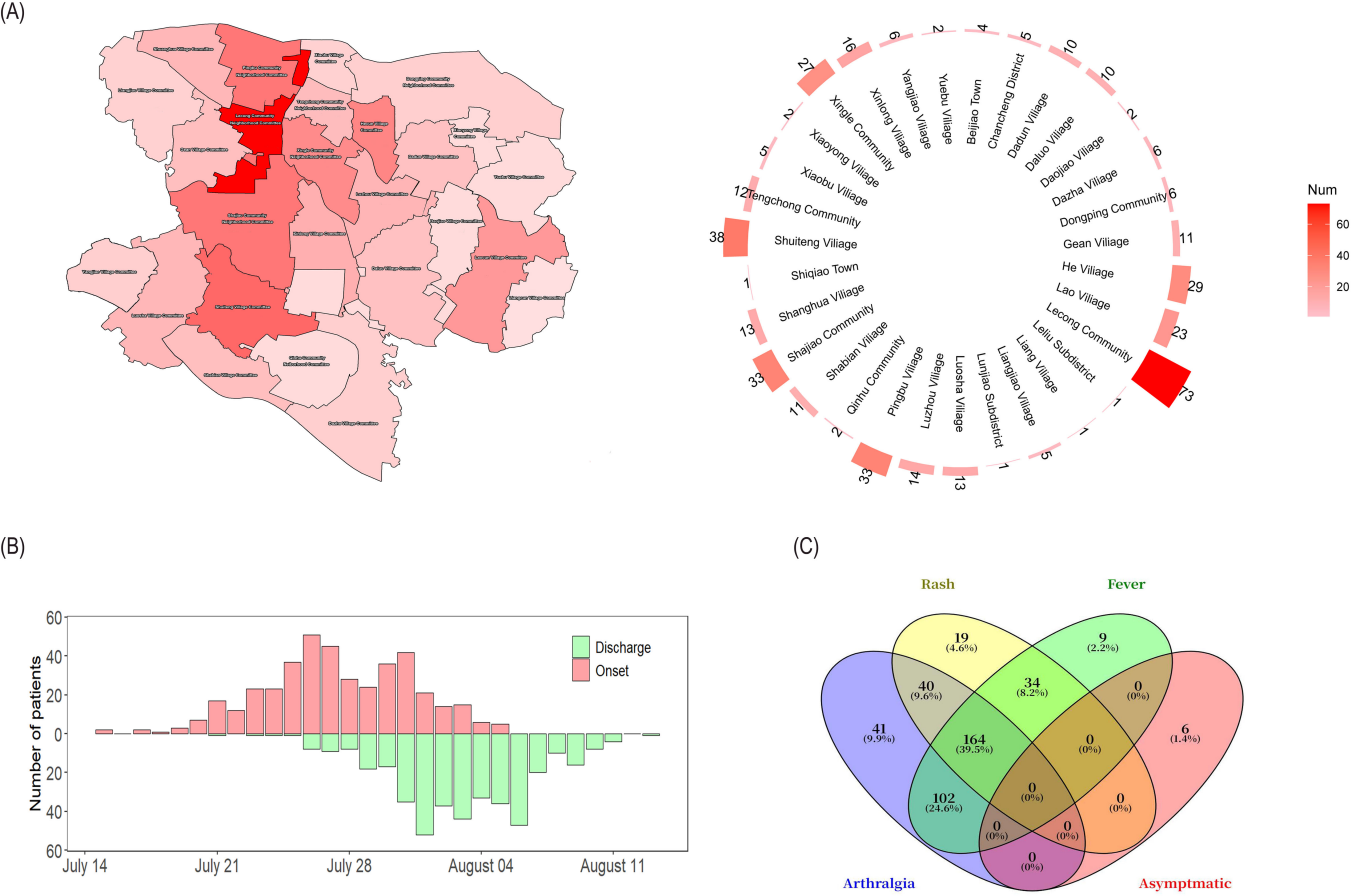
Epidemiological and clinical characteristics of CHIKF cases in Shunde, Foshan, 2025. **(A)** Spatial distribution of confirmed CHIKF cases in Shunde. **(B)** Temporal distribution of cases by date of symptom onset and hospital discharge. **(C)** Distribution of clinical symptom—rash, fever, and arthralgia and asymptomatic—in CHIKV cases. The numbers and percentages in Venn diagram to indicate the count and proportion of patients exhibiting each symptom combination.

A total of 415 laboratory-confirmed cases of acute CHIKV infection were recorded during the study period. Cases were confined to a 20-day outbreak window (July 15 - August 13, 2025) and originated from four administrative divisions in Lecong, Shunde, Foshan: Lecong Community Neighborhood Committee (73 cases, 17.59%), Shuiteng Village Committee (38 cases, 9.16%), Shajiao Community Neighborhood Committee (33 cases, 7.95%), and Pingbu Community Neighborhood Committee (33 cases, 7.95%) (**Figure 1A**, 1B). The average disease duration was 5.65 days, and discharge dates were predominantly clustered between July 21 and August 13 (**Figure 1B**). Sociodemographic analysis revealed a slight female predominance (54.22% female, 45.78% male), with the 46–65 years age group representing the largest proportion of cases (130 patients, 31.33%). Most patients (79.28%) had no pre-existing chronic conditions; among those with comorbidities, hypertension (9.89%), chronic gastritis (5.30%), and diabetes (3.61%) were the most common (**Table 1**).

**Table 1:** Baseline and clinical characteristics of the study population in patients with Chikungunya fever.

Variables	Characteristics	N(%)
Age	0-6	26 (6.27)
	7-18	89 (21.45)
	19-45	115 (27.71)
	46-65	130 (31.33)
	≥66	55 (13.25)
Gender	Male	190 (45.78)
	Female	225 (54.22)
Presence of Chronic Disease	None	329 (79.28)
	Hypertension	41 (9.89)
	Chronic gastritis/Stomach ulcer	22 (5.30)
	Diabetes	15 (3.61)
	Rhinitis	8 (1.93)
	Hyperlipidemia	7 (1.69)
	Hepatitis	5 (1.20)
	Cardiovascular disease	4 (0.96)
	Others	12 (2.89)
Clinical features	Arthralgia	347 (83.61)
	Fever	309 (74.46)
	Rash	257 (61.93)
	Fatigue	42 (10.12)
	Aphthous ulcer	37 (8.92)
	Conjunctival injection	16 (3.86)
	Headache	15 (3.61)
	Diarrhea	14 (3.37)
	Nausea	12 (2.89)

### 3.2 Joint pain, fever, and rash are the main clinical manifestations in CHIKF patients

Asymptomatic infections were the least frequent (1.45%). Regarding the resolution of these main symptoms, the median time for fever resolution was 1 (IQR 0, 2) day, for arthralgia relief was 3 (IQR 2, 4) days, and for rash resolution was 3 (IQR 2, 4) days. A Venn diagram illustrates the distribution of different combinations of main symptoms among the patient population (**Figure 1C**). Among 415 confirmed acute CHIKF cases, the main clinical manifestations were, in order, arthralgia (83.61%), fever (74.46%), and rash (61.93%) (**Table 1**). A small number of patients had sporadic symptoms including fatigue (10.12%), oral ulcer (8.92%), conjunctival congestion (3.86%), headache (3.61%), diarrhea (3.37%), and nausea (2.89%). Among these symptoms, arthralgia primarily affected the ankle (42.89%), wrist (32.53%), and finger joints (32.29%); fever mainly presented as low-grade or moderate (< 39°C), with only 7.23% of patients exhibiting high fever (≥ 39°C); and the rash primarily appeared as maculopapular (37.59%) and mainly affected the limbs (50.12%) (**Supplementary Table S1**).

Among these patients, those presenting with two of the three main symptoms constituted the largest proportion (42.41%). The combination of arthralgia + fever was the most common (24.58%), followed by similar proportions for arthralgia + rash (9.64%) and rash + fever (8.19%). Patients presenting with all three main symptoms were the next largest group (39.52%). Patients with only a single symptom accounted for only 16.63% of the total, with arthralgia being the most common single symptom (9.88%), followed by rash (4.58%) and fever (2.17%).

### 3.3 Logistic regression analysis of clinical data in CHIKF patients grouped by symptom burden

Based on the distribution characteristics of symptom burden, patients were divided into four groups according to the number of main symptoms present: asymptomatic group (6 individuals), single-symptom group (69 individuals), double-symptom group (176 individuals), and triple-symptom group (164 individuals). Univariate analysis was initially used to assess the distribution characteristics of a series of clinical data, including demographics and hematological indicators, across these symptom groups.

Demographic data presented as frequency/percentage and quartiles (**Table 2**) showed that age exhibited a significant distribution difference among the symptom groups (H = 36.721, *P* < 0.001). The asymptomatic group was relatively evenly distributed across age groups, while the single- and double-symptom groups were more common in patients aged 46–65 years. In contrast, the triple-symptom group was distinctly concentrated in patients aged 7–45 years, showing a typical clustered distribution. Although the number of females was slightly higher than males in each symptom group, gender did not show a differential distribution across the groups. While only 20.72% of patients had various chronic medical histories, the distribution of patients with such histories still showed a statistically significant difference among symptom groups (Z = -2.663, *P* = 0.008). Regarding body temperature, patients in different symptom groups also showed different distribution patterns (H = 176.501, *P* < 0.001). The vast majority in the single-symptom group had normal temperature, with patients presenting primarily with isolated rash or arthralgia. Only 9 patients had low or moderate fever. In the double-symptom group, the distribution of patients with low and moderate fever was similar and highest, with a small number having high fever. In the triple-symptom group, patients most frequently had moderate fever, followed by low fever, with only a minority having high fever. The duration of fever also showed different distribution patterns across symptom groups. In pairwise comparisons, except for no statistical difference between the asymptomatic and single-symptom groups, all other inter-group comparisons showed significant differences, displaying a trend of increasing fever duration with the number of symptoms (H = 119.413, *P* < 0.001).

**Table 2:** Distribution and association of symptom burden in demographic characteristics.

Characteristics	Asymptomatic group, (n=6)	Single-symptom group, (n=69)	Double-symptom group, (n=176)	Triple-symptom group, (n=164)	Z/H	*P* value
Age					36.721	< 0.001
0-6	0 (0.00)	2 (0.48)	10 (2.41)	14 (3.37)		
7-18	1 (0.24)	10 (2.41)	28 (6.75)	50 (12.05)		
19-45	1 (0.24)	15 (3.61)	42 (10.12)	57 (13.73)		
46-65	4 (0.96)	26 (6.27)	64 (15.42)	36 (8.67)		
>65	0 (0.00)	16 (3.86)	32 (7.71)	7 (1.69)		
Gender					-0.688	0.492
Male	2 (0.48)	36 (8.67)	79 (19.04)	73 (17.59)		
Female	4 (0.96)	33 (7.95)	97 (23.37)	91 (21.93)		
Chronic disease					-2.663	0.008
No	4 (0.96)	54 (13.01)	128 (30.84)	143 (34.46)		
Yes	2 (0.48)	15 (3.61)	48 (11.57)	21 (5.06)		
Temperature					176.501	< 0.001
Normal	6 (1.45)	60 (14.46)	40 (9.64)	0 (0.00)		
Low-grade fever	0 (0.00)	5 (1.20)	61 (14.70)	59 (14.22)		
Moderate fever	0 (0.00)	4 (0.96)	62 (14.94)	88 (21.20)		
High fever	0 (0.00)	0 (0.00)	13 (3.13)	17 (4.10)		
Duration of fever^a^	0 (0, 0)	0 (0, 0)	1 (0.5, 2)	1 (1, 2)	119.413	< 0.001
Nucleic acid conversion times^a^	6 (5, 7)	5 (4, 6)	5 (5, 6)	6 (5, 6)	5.973	0.113

Data are n (%) unless otherwise indicated.

IQR, interquartile range.

a Data are median (IQR).

Hematological indicators presented as quartiles (**Supplementary Table S2**) showed that routine blood tests, liver and kidney function also exhibited different distribution patterns across symptom groups. Leukocytes count, neutrophil (NE) count, platelet (PLT) count, and estimated glomerular filtration rate (eGFR) showed a gradual increasing trend with the increasing number of symptoms, with statistical differences between groups (*P* < 0.05). In contrast, blood urea nitrogen and creatinine (CR) showed a gradually decreasing trend with increasing symptom number, also with statistical differences between groups (*P* < 0.05). Interestingly, CRP showed a characteristic trend of first increasing and then decreasing as the number of symptoms increased, also with statistical differences between groups (*P* < 0.05). Other indicators showed no significant abnormalities.

To further characterize and identify independent risk factors associated with symptom burden, the asymptomatic and single-symptom groups were classified as the lower burden group, and the double-symptom and triple-symptom groups as the higher burden group. Multivariate logistic regression analysis was performed to construct a model. The results found that among demographic data, age (OR = 0.979, 95% CI: 0.960-0.998, *P* = 0.029), gender (OR = 0.528, 95% CI: 0.289-0.965, *P* = 0.038), and fever duration (OR = 8.156, 95% CI: 4.544-14.639, *P* < 0.001) were included in the model (**Table 3**). Older age (per 1-year increase) and male gender (reference to female) were protective factors, and negatively correlated with high symptom burden, while longer fever duration was a risk factor associated with a higher symptom burden. Among hematological indicators, only eGFR was included in the model (OR = 1.008, 95% CI: 1.000-1.016, *P* = 0.005). A lower eGFR was a risk factor associated with higher symptom burden (**Supplementary Table S3**).

**Table 3:** Multivariate logistic regression analysis of demographic characteristics associated with symptom burden.

Characteristics	Multivariate	
	OR (95%*CI*)	*P* value
Age	0.979 (0.960-0.998)	0.029
Gender	0.528 (0.289-0.965)	0.038
Hypertension	1.211 (0.252-5.814)	0.811
Hyperlipidemia	0.223 (0.025-1.965)	0.177
Diabetes	1.089 (0.178-6.683)	0.926
Chronic gastritis/Peptic ulcer	0.395 (0.050-3.136)	0.379
Duration of fever	8.156 (4.544-14.639)	< 0.001
Nucleic acid conversion times	0.968 (0.796-1.178)	0.748

### 3.4 Logistic regression analysis of clinical data in CHIKF patients grouped by fever severity

Patients were divided into four groups based on fever severity: normal group (106 individuals), low-grade fever group (125 individuals), moderate fever group (154 individuals), and high fever group (30 individuals). Univariate analysis was initially used to assess the distribution characteristics of a series of clinical data, including demographics and hematological indicators, across these fever severity groups.

Demographic data presented as frequency/percentage and quartiles (**Table 4**) showed that age again showed a significant statistical difference across the fever severity groups (H = 42.584, *P* < 0.001). The normal temperature group consisted mostly of patients aged 46–65 years. The low-grade and moderate fever groups consisted mostly of patients aged 19–65 years. The high fever group consisted mostly of students aged 7–18 years. The number of symptoms also showed a statistical difference across fever severity groups (H = 147.639, *P* < 0.001). Patients in the normal temperature group primarily had isolated arthralgia or rash, or both. All febrile patients were typically accompanied by arthralgia or rash, or both. In the low-grade and high fever groups, the numbers of patients with two and three symptoms were similar, while the moderate fever group was characteristically represented by patients with three symptoms. The duration of fever also showed different distribution patterns across the fever severity groups (H = 249.866, *P* < 0.001). In pairwise comparisons, statistical differences existed only between the low-grade and high fever groups, and between the normal temperature group and all fever groups.

**Table 4:** Distribution and association of the height of fever across demographic characteristics.

Characteristics	Normal group, (n=106)	Low-grade fever group, (n=125)	Moderate fever group, (n=154)	High fever group, (n=30)	Z/H	*P* value
Age					42.584	< 0.001
0-6	1 (0.24)	9 (2.17)	13 (3.13)	3 (0.72)		
7-18	16 (3.86)	25 (6.02)	35 (8.43)	13 (3.13)		
19-45	27 (6.51)	40 (9.64)	44 (10.60)	4 (0.96)		
46-65	41 (9.88)	38 (9.16)	42 (10.12)	9 (2.17)		
>65	21 (5.06)	13 (3.13)	20 (4.82)	1 (0.24)		
Gender					-1.887	0.059
Male	44 (10.60)	52 (12.53)	77 (18.55)	17 (4.10)		
Female	62 (14.94)	73 (17.59)	77 (18.55)	13 (3.13)		
Chronic disease					-0.079	0.937
No	81 (19.52)	104 (25.06)	122 (29.40)	22 (5.30)		
Yes	25 (6.02)	21 (5.06)	32 (7.71)	8 (1.93)		
Symptom					147.639	< 0.001
Asymptomatic	6 (1.45)	0 (0.00)	0 (0.00)	0 (0.00)		
Single-symptom	60 (14.46)	5 (1.20)	4 (0.96)	0 (0.00)		
Double-symptom	40 (9.64)	61 (14.70)	62 (14.94)	13 (3.13)		
Triple-symptom	0 (0.00)	59 (14.22)	88 (21.20)	17 (4.10)		
Duration of fever^a^	0 (0, 0)	1 (1, 2)	2 (1, 2)	2 (1.75, 3)	249.866	< 0.001
Nucleic acid conversion times^a^	5 (4, 6)	6 (5, 6)	6 (5, 6)	6 (5, 7)	7.117	0.068

Data are n (%) unless otherwise indicated.

IQR, interquartile range.

a Data are median (IQR).

Hematological indicators presented as quartiles (**Supplementary Table S4**) showed that routine blood tests, liver and kidney function also exhibited different distribution patterns across the fever severity groups. Leukocytes count, NE count, and eGFR showed some increasing trend between the normal temperature group and the fever groups, while blood urea nitrogen and CR showed a decreasing trend between the normal temperature group and the fever groups. However, there were no significant differences among the different fever groups themselves, suggesting that changes in these routine laboratory indicators are likely due to elevated body temperature, disruption of internal homeostasis, and immune system activation.

Similarly, to further characterize and identify independent risk factors associated with symptom severity, the normal temperature and low-grade fever groups were classified as the mild group, and the moderate and high fever groups as the severe group. Multivariate logistic regression analysis was performed to construct a model. The results found that among demographic data, only symptom burden (OR = 10.198, 95% CI: 3.257-31.933, *P* < 0.001) and fever duration (OR = 2.591, 95% CI: 1.970-3.409, *P* < 0.001) were included in the model (**Table 5**). Both multiple symptoms and longer fever duration were risk factors associated with fever severity. Among hematological indicators, only CR (OR = 1.041, 95% CI: 1.014-1.069, *P* = 0.003) and eGFR (OR = 1.043, 95% CI: 1.025-1.062, *P* < 0.001) were included in the model (**Supplementary Table S5**). Lower CR levels and higher eGFR were both risk factors associated with fever severity.

**Table 5:** Multivariate logistic regression analysis of demographic characteristics across the fever severity groups.

Characteristics	Multivariate	
	OR (95%CI)	*P* value
Age	0.993 (0.979-1.007)	0.339
Gender	1.383 (0.858-2.229)	0.182
Hypertension	1.096 (0.325-3.693)	0.882
Hyperlipidemia	0.370 (0.029-4.727)	0.445
Diabetes	0.594 (0.143-2.456)	0.472
Chronic gastritis/Peptic ulcer	1.007 (0.168-6.028)	0.994
Clinical symptoms	10.198 (3.257-31.933)	< 0.001
Duration of fever	2.591 (1.970-3.409)	< 0.001
Nucleic acid conversion times	1.098 (0.928-1.300)	0.277

### 3.5 Logistic regression analysis of clinical data in CHIKF patients grouped by nucleic acid conversion time

**Figure 2:**
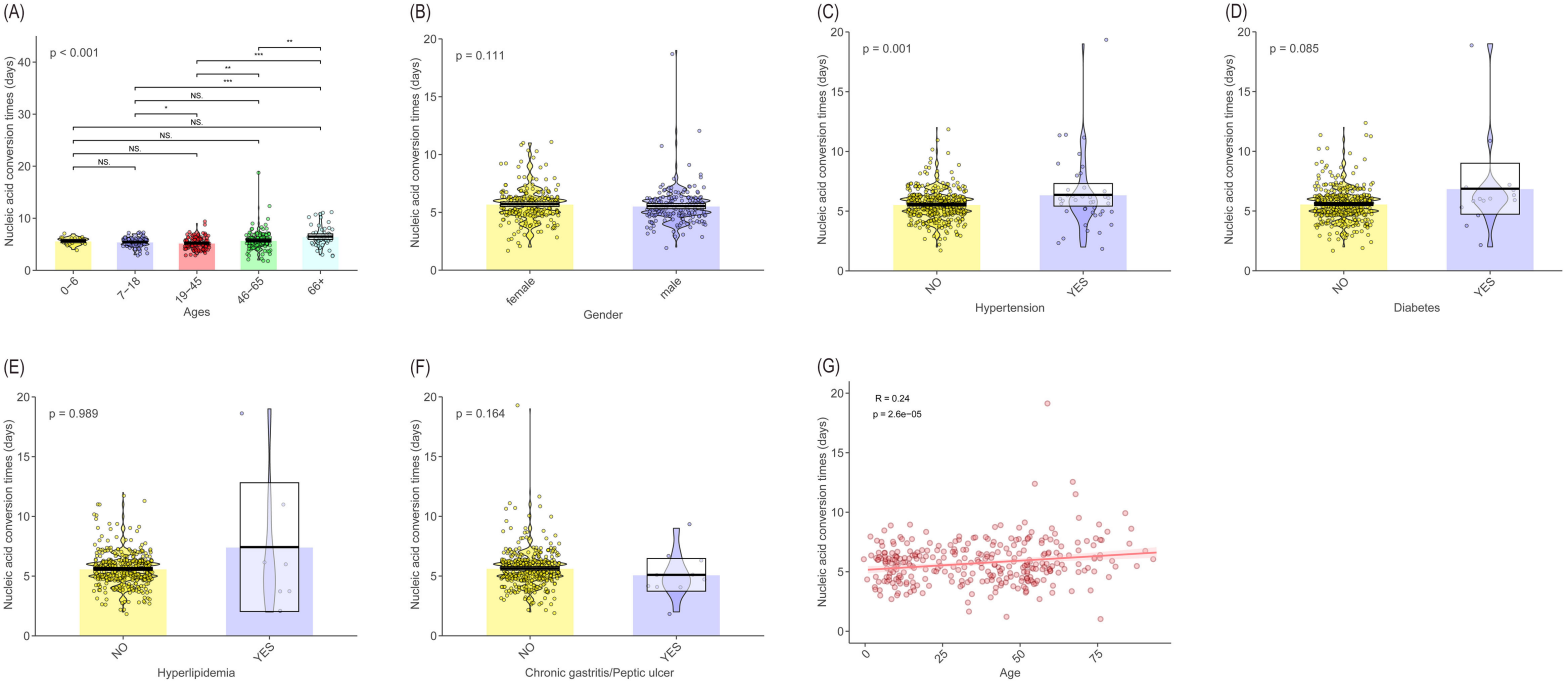
Associations between disease duration and clinical/demographic variables. **(A-G)** Pirate plot comparing disease duration by **(A)** age group, **(B)** gender, **(C)** hypertension, **(D)** diabetes, **(E)** hyperlipidemia, and **(F)** chronic gastritis/peptic ulcer. *P* values were calculated using the Mann-Whitney U test (for two groups) or Kruskal-Wallis test (for > 2 groups). **(G)** Spearman correlation analysis between age and disease duration. The coefficient *R* and *P* value are shown. Significance is defined as * *P* < 0.05, ** *P* < 0.01, and *** *P* < 0.001.

Subsequently, we focused on the time to nucleic acid conversion (negative result). Comparing demographic data across different categories revealed that older patients had a significantly longer disease duration than younger individuals (*P* < 0.001). There was a weak but significant positive correlation between age and disease duration (R = 0.24, *P* = 2.6e-05). Similarly, the presence of hypertension was also significantly associated with a prolonged clinical course (*P* = 0.001). In contrast, no significant differences were observed based on gender (*P* = 0.111), diabetes (*P* = 0.085), hyperlipidemia (*P* = 0.989), or history of chronic gastritis/peptic ulcer (*P* = 0.164) (**Figure 2**).

Meanwhile, using the median nucleic acid conversion time of 6 (IQR 5, 6) days, the clinical data were divided into a rapid conversion group (≤6 days) and a slow conversion group (> 6 days). Logistic regression analysis was performed to construct a model. Age (OR = 1.022, 95% CI: 1.012-1.030, *P* < 0.001), PLT (OR = 0.992, 95% CI: 0.987-0.996, *P* < 0.001), and eGFR (OR = 0.990, 95% CI: 0.987-0.998, *P* = 0.046) were included in the regression equation. Older age was an independent risk factor affecting the nucleic acid conversion time, while increased PLT and increased eGFR were independent protective factors affecting the nucleic acid conversion time (**Supplementary Table S6**-**Supplementary Table S7**).

In summary, older age and male gender are independent protective factors affecting the number of main clinical symptoms in CHIKF patients, while longer fever duration and lower eGFR are independent risk factors affecting the number of main clinical symptoms. Secondly, greater symptom burden, longer fever duration, lower CR levels, and relatively higher eGFR are independent risk factors affecting the fever severity in CHIKF patients. Thirdly, older age is an independent risk factor affecting the nucleic acid conversion time in CHIKF patients, while relative increases in PLT and eGFR are protective factors.

### 3.6 Genomic analysis links outbreak strains to Réunion Island lineage

For genomic characterization of CHIKV, we selected 92 samples having nucleic acid Ct values of less than 30 for targeted whole-genome sequencing, resulting in 92 complete genome sequences. All sequences were deposited in the GenBase database of CNCB (China National Center for Bioinformation) under accession number C_AA119371.1 - C_AA119462.1.

**Figure 3:**
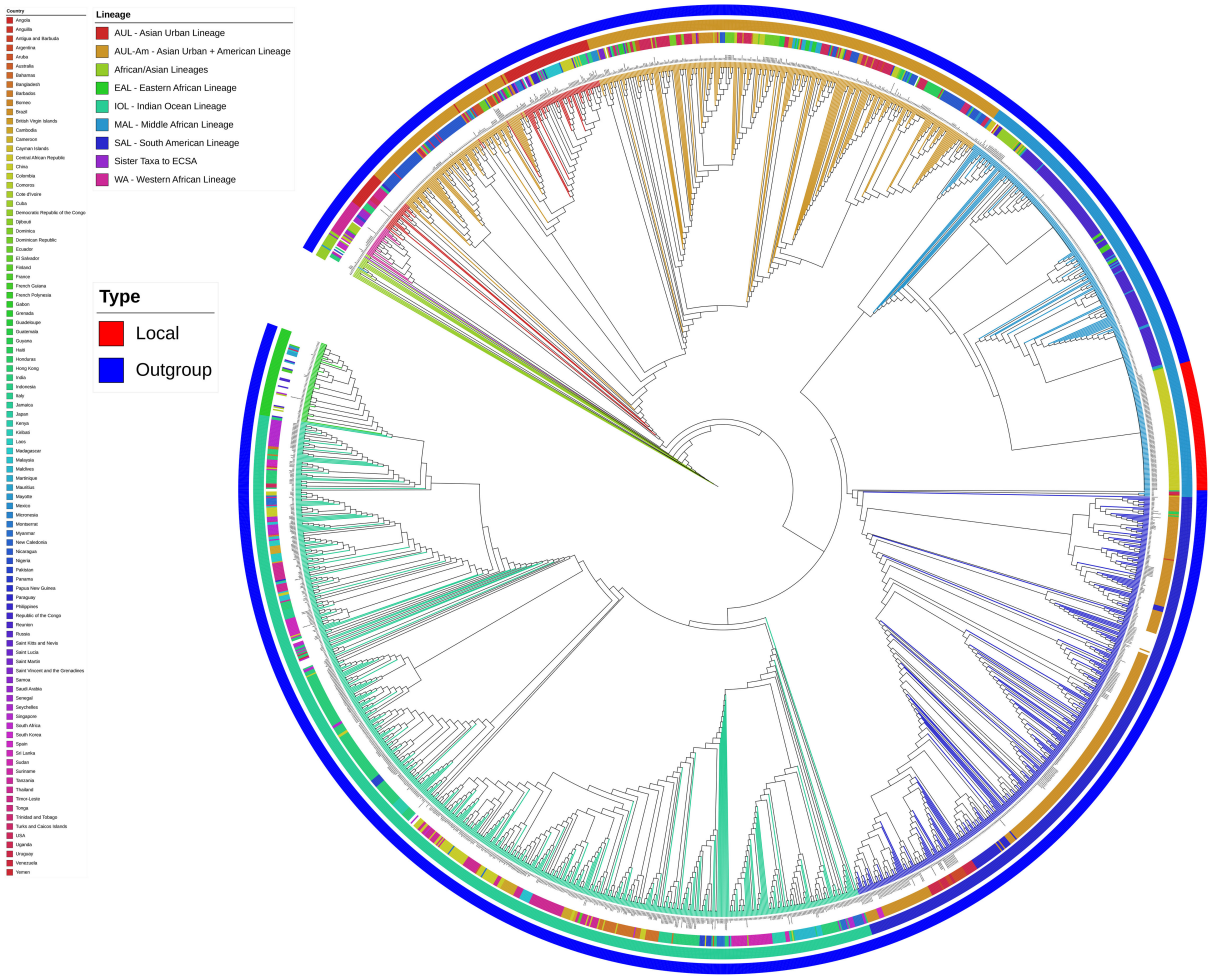
Phylogenetic analysis of global CHIKV. From inner to outer rings: viral lineage, country of origin, and strain type. Local outbreak strains are highlighted labeled in red on the outermost ring.

Phylogenetic analysis revealed that all the outbreak strains belonged to the East/Central/South African (ECSA) lineage, specifically clustering within MAL (**Figure 3**). The closest related strain within the same clade originated from the 2025 CHIKV outbreak on Réunion Island, France (99.96%-100% nucleotide similarity).

Mutation analysis identified between one and six mutations per strain relative to the Réunion Island reference strain 80652-1-1-1 (GenBank: PV700165.1). This included one shared synonymous mutation (C3876T). Two additionally clustered mutations—G7431A and T10430C—were detected in 29.35% (27/92) and 13.04% (12/92) of sequences, respectively (**Supplementary Figure S1**). Neither mutation resulted in amino acid change.

Importantly, all strains this from outbreak carried two previously reported adaptive mutations, E1-A226V and E2-I211T, both of which have been implicated in enhanced transmission efficiency by Aedes albopictus mosquitoes (Frumence et al, 2025).

## 4 Discussion

This study provides a comprehensive characterization of the 2025 CHIKF outbreak in Foshan—recognized as the largest localized CHIKF outbreak in Guangdong Province to date. By systematically analyzing the clinical spectrum, laboratory profiles, and determinants of symptom burden and disease duration, the research reveals crucial epidemiological and pathophysiological features of CHIKV infection in this endemic population.

CHIKV infection was distributed across all age groups, with the highest proportion observed in the 46–65 years cohort (31.33%), and no significant gender disparity was detected. This demographic pattern aligns with the majority of global CHIKV outbreak reports (Amin et al, 2022; Chis Ster et al, 2020; Tun et al, 2022), reflecting the virus’s broad infectivity across age strata and the absence of gender transmission biases. Clinically, the classic triad of symptoms—arthralgia (83.61%), fever (74.46%), and rash (61.93%)—predominated, aligning with international findings on acute CHIKV infection (Babu et al, 2025; Tavares et al, 2025). Severe arthralgia emerged as a defining characteristic, with the wrists, ankles, and fingers being the most commonly affected joints; this mirrors prior observations that CHIKV-associated arthralgia typically presents as symmetrical polyarthropathy involving both small and large peripheral joints (Khongwichit et al, 2021). Cutaneous manifestations were primarily maculopapular rash (37.59%), with a predilection for the limbs (50.12%), congruent with reports of CHIKV-associated exanthems that often distribute symmetrically over extensor surfaces (Gonçalves Maciel et al, 2022; Lozano-Parra et al, 2024). Additionally, less common symptoms (fatigue: 10.12%; aphthous ulcers: 8.92%; conjunctival injection: 3.86%) were documented. This overall pattern, however, presents an interesting context for the genomic finding of an epidemic strain harboring adaptive mutations (E1-A226V, E2-I211T), suggesting a potential dissociation between its transmission efficiency and clinical severity in this setting. These findings highlight the need for more detailed surveillance data to fully capture the symptom spectrum of CHIKF, as well as heightened clinical awareness of atypical presentations to improve early case identification and facilitate differential diagnosis from other febrile illnesses (e.g; dengue, Zika).

Multivariate logistic regression analyses identified key predictors of CHIKF outcomes: younger age and female gender were associated with a higher likelihood of presenting with multiple symptoms, while advanced age correlated with prolonged disease duration. The association between older age and delayed recovery aligns with established concepts of immunosenescence—where age-related declines in innate and adaptive immune function impair viral clearance in arboviral infections (Cerqueira-Silva et al, 2024; Lang et al, 2017). Among blood biomarkers, renal function indicators—particularly eGFR—exerted a substantial influence, demonstrating significant associations with symptomatic presentation, fever severity, and disease duration. Inflammatory markers also exhibited notable differences across symptom groups: NE counts (H = 14.217, *P* = 0.003) and CRP levels (H = 16.401, *P* = 0.001) were elevated in patients with more symptoms. NE are key effectors in the acute immune response to CHIKV, migrating to infection sites to produce reactive oxygen species (ROS) and pro-inflammatory mediators that support viral clearance; elevated CRP, a marker of systemic inflammation, further confirms robust immune activation—consistent with previous reports of heightened inflammatory responses in acute CHIKV infection (Henderson Sousa et al, 2023). Lymphocytopenia, a hallmark of a potent innate immune activation against the virus (Lum et al, 2024; Rudd et al, 2021), was not observed in this cohort. This discrepancy is likely attributable to the mild disease severity of most cases, as lymphocytopenia is more commonly associated with severe or complicated CHIKV infection. Importantly, while acute inflammation is critical for viral clearance, it also serves as a primary driver of clinical symptoms such as arthralgia and fever (Fox and Diamond, 2016; Rodríguez-Morales et al, 2016).

Currently, data on the relationship between laboratory indicators and clinical outcomes in CHIKF remain limited. However, insights from studies of COVID-19 and dengue fever—two febrile illnesses with overlapping epidemiological and clinical features—offer valuable parallels. In COVID-19, laboratory abnormalities including elevated D-dimer, liver enzymes, CR, urea, and CRP, as well as decreased lymphocyte and PLT counts, have been correlated with adverse outcomes (Alsayed et al, 2024). Similarly, in dengue virus infection, thrombocytopenia, leukopenia, and elevated liver enzymes are common laboratory abnormalities associated with severe disease (Mahmood et al, 2021). These studies demonstrate that laboratory markers (eGFR, PLT) can serve as prognostic tools to identify high-risk patients, guide clinical decision-making, and enable targeted interventions—insights that are extendable to CHIKF management.

Beyond clinical and laboratory analyses, characterizing the genetic variation of circulating CHIKV strain is critical for outbreak source tracing and epidemic control. Whole-genome sequencing and comparative analysis with historical CHIKV strains revealed that the Shunde outbreak strains belong to MAL. Furthermore, these strains exhibited high sequence homology with isolates from the 2025 CHIKV outbreak in Réunion Island, France—suggesting a potential epidemiological link between the two outbreaks possibly through a common ancestral source or direct transmission. The presence of shared mutations (G7431A, T10430C) further supports a common transmission lineage. Previous studies have established that specific CHIKV mutations enhance vector adaptability: the E1-A226V substitution increases viral infectivity in *Aedes albopictus* midgut cells (Nadim Sharif et al, 2021), while the E2-I211T mutation provides a favorable genetic background that potentiates the transmission advantage of E1-A226V (and the E2-L210Q mutation is associated with enhanced viral dissemination) (Frumence et al, 2025). In this study, the Shunde outbreak strains harbored both E1-A226V and E2-I211T. Given that *Aedes albopictus* is the primary mosquito vector in Shunde, these adaptive mutations likely enhanced viral transmission efficiency, facilitating the rapid emergence of autochthonous CHIKF cases during the outbreak.

Notably, while the ECSA lineage has been epidemiologically associated with outbreaks characterized by prominent arthralgia and significant febrile morbidity (Bettis et al, 2022; Sharif et al, 2021), the MAL strains identified in this outbreak exhibited an apparent dissociation between clinical severity and transmission dynamics. Despite demonstrating enhanced transmissibility, potentially facilitated by the *Aedes albopictus*-adaptive mutations E1-A226V and E2-I211T, these strains were associated with a clinically attenuated phenotype. This was evidenced by significantly lower rates of high-grade fever and reduced overall symptom complexity. Such discordance suggests that while specific adaptive mutations can increase viral fitness in mosquito vectors, additional viral genetic factors or host-specific factors may concurrently temper disease manifestation, underscoring the complex interplay between viral evolution, transmission efficiency, and clinical outcome in emerging CHIKV outbreaks.

The retrospective design introduces selection bias and limits generalizability, the study provides clinically useful prognostic markers and emphasizes monitoring hematological/inflammatory parameters—especially in older adults. It also highlights the need for enhanced molecular surveillance to understand transmission dynamics and trace outbreak sources. In conclusion, systematic CHIKV outbreak data collection is essential for developing effective control and prevention strategies.

## 5 Conclusion

This comprehensive characterization of a major CHIKF outbreak identifies distinct clinical and laboratory predictors of symptom burden and disease duration. Furthermore, genomic analysis reveals the circulation of an adapted viral strain exhibiting enhanced transmission efficiency. Notably, the dissociation between high transmissibility and mild clinical manifestations observed in this outbreak underscores the critical need for integrated clinical and molecular surveillance systems to effectively monitor and control emerging arboviral disease in southern China.

## Data Availability

The sequence data have been deposited in the GenBase database of the National Genomic Data Center with the accession numbers C_AA119371.1 to C_AA119462.1. The data are publicly accessible via https://ngdc.cncb.ac.cn/genbase.

## Supplementary material

Supplementary Figure 1 Mutation sites identified in the local outbreak strains. The reference genome is strain 80652-1-1-1. The horizontal axis indicates the genome positions of observed mutations relative to the reference sequence.
