# Supplementary material for: Clinical epidemiology and viral genomics insights from a Chikungunya fever outbreak in South China, 2025
Source: Front Cell Infect Microbiol. 2026 Jan 23;16:1762631. doi: 10.3389/fcimb.2026.1762631 (PMC12876165; doi:10.3389/fcimb.2026.1762631)
Supplement: Supplementary file 2 [file Table1.docx]

Supplementary materials

**Table S1** Common fever pattern, skin rash sites and joint sites in Chikungunya fever patients.

| **Clinical features** | **N** | **Percentage (%)** |
| --- | --- | --- |
| **Fever** |  |  |
| Low-grade fever | 125 | 30.12 |
| Moderate fever | 154 | 37.11 |
| High fever | 30 | 7.23 |
| **Skin rash** |  |  |
| Maculopapular rash | 156 | 37.59 |
| morbilliform | 42 | 10.12 |
| Petechiae | 33 | 7.95 |
| Trunk | 154 | 37.11 |
| Limbs | 208 | 50.12 |
| Face | 53 | 12.77 |
| Palm/Sole | 13 | 3.13 |
| **Joint pain** |  |  |
| Finger | 134 | 32.29 |
| Wrist | 135 | 32.53 |
| Elbow | 43 | 10.36 |
| Shoulder | 28 | 6.75 |
| Toes | 60 | 14.46 |
| Ankle | 178 | 42.89 |
| Knee | 91 | 21.93 |
| Hip | 10 | 2.41 |
| Spine | 7 | 1.69 |

**Table S2** Distribution and association of clinical symptoms in blood test, liver function test and kidney function test.

| **Characteristics** | **Asymptomatic group** | **Single-symptom group** | **Double-symptom group** | **Triple-symptom group** | **H** | ***P* value** |
| --- | --- | --- | --- | --- | --- | --- |
| **Blood test^a^** |  |  |  |  |  |  |
| Leukocytes (10^9^/L) | 4.23 (3.57-4.49) | 5.18 (3.94-6.14) | 5.59 (4.34-6.88) | 5.56 (4.99-6.80) | 13.524 | 0.004 |
| NE (10^9^/L) | 2.55 (1.91-2.77) | 3.56 (2.55-4.77) | 4.11 (3.01-5.38) | 4.18 (3.31-5.15) | 14.217 | 0.003 |
| LY (10^9^/L) | 1.20 (0.84-1.26) | 0.93 (0.61-1.16) | 0.83 (0.64-1.19) | 0.89 (0.63-1.22) | 1.591 | 0.661 |
| MO (10^9^/L) | 0.42 (0.29-0.50) | 0.46 (0.36-0.6) | 0.44 (0.37-0.59) | 0.48 (0.37-0.58) | 3.106 | 0.376 |
| HGB (g/L) | 129.00 (120.75-147.00) | 134.00 (125.25-146.00) | 133.00 (124.00-144.00) | 135.00 (124.00-145.00) | 0.352 | 0.950 |
| PLT (10^9^/L) | 170.50 (159.75-182.75) | 203.50 (173.75-239.00) | 206.00 (172.00-251.00) | 225.00 (187.75-265.50) | 9.856 | 0.020 |
| CRP (mg/L) | 4.24 (0.60-12.20) | 10.37 (5.40-16.95) | 11.81 (5.26-26.65) | 5.48 (2.69-13.31) | 16.401 | 0.001 |
| **Liver function test^b^** |  |  |  |  |  |  |
| ALT (U/L) | 20.50 (16.88-23.00) | 22.70 (15.40-27.80) | 19.40 (14.35-28.75) | 18.30 (13.90-28.50) | 2.062 | 0.560 |
| AST (U/L) | 22.45 (18.65-25.28) | 25.30 (21.4-33.15) | 24.10 (20.80-31.18) | 26.10 (20.20-34.20) | 2.358 | 0.501 |
| TBIL (μmol/L) | 9.35 (6.35-11.68) | 8.10 (5.73-10.90) | 8.65 (6.50-12.48) | 8.35 (6.00-11.45) | 3.593 | 0.309 |
| DBIL (μmol/L) | 4.05 (3.33-4.55) | 3.70 (3.00-4.50) | 3.70 (3.00-5.08) | 3.60 (2.88-4.60) | 2.648 | 0.449 |
| IBIL (μmol/L) | 5.20 (3.18-7.23) | 4.35 (2.80-6.48) | 4.95 (3.50-7.55) | 4.60 (2.98-7.00) | 4.701 | 0.195 |
| **Kidney function test^c^** |  |  |  |  |  |  |
| Urea (mmol/L) | 5.40 (4.53-6.99) | 5.00 (3.85-6.30) | 4.57 (3.87-5.50) | 4.20 (3.50-4.90) | 20.413 | < 0.001 |
| UA (μmol/L) | 321.00 (303.25-380.00) | 291.00 (240.50-369.50) | 293.00 (247.25-346.75) | 283.00 (242.00-337.00) | 4.009 | 0.261 |
| CR (μmol/L) | 68.50 (68.00-72.75) | 64.00 (50.50-78.00) | 62.00 (47.25-77.00) | 52.50 (42.75-67.25) | 18.840 | < 0.001 |
| CO_2_ (mmol/L) | 23.65 (23.15-24.00) | 24.05 (22.90-25.50) | 24.00 (22.60-25.50) | 23.70 (22.25-25.00) | 2.891 | 0.409 |
| eGFR (mL/min) | 82.86 (79.08-116.93) | 100.46 (87.73-117.81) | 103.22 (88.97-120.54) | 119.37 (105.21-156.48) | 37.313 | < 0.001 |

^a^ NE: Neutrophils; LY: Lymphocytes; MO: Monocytes; HGB: Hemoglobin; PLT: Platelets; CRP: C-reactive protein.

^b^ ALT: Alanine aminotransferase; AST: Aspartate aminotransferase; TBIL: Total bilirubin; DBIL: Direct bilirubin; IBIL: Indirect bilirubin.

^c^ UA: Uric acid; CR: Creatinine; eGFR: estimated glomerular filtration rate.

**Table S3** Multivariate logistic regression analysis of blood test, liver function test and kidney function test associated with minor-symptoms and multi-symptoms.

| **Characteristics** | **Multivariate** | |
| --- | --- | --- |
|  | **OR (95%*CI*)** | ***P* value** |
| **Blood test^a^** |  |  |
| Leukocytes (10^9^/L) | 1.186 (0.989-1.422) | 0.183 |
| NE (10^9^/L) | 1.222 (1.009-1.480) | 0.188 |
| LY (10^9^/L) | 0.928 (0.537-1.601) | 0.296 |
| MO (10^9^/L) | 1.603 (0.315-8.162) | 0.091 |
| HGB (g/L) | 1.012 (0.993-1.032) | 0.451 |
| PLT (10^9^/L) | 1.001 (0.996-1.006) | 0.982 |
| CRP (mg/L) | 1.009 (0.987-1.032) | 0.305 |
| **Liver function test^b^** |  |  |
| ALT (U/L) | 1.010 (0.991-1.029) | 0.057 |
| AST (U/L) | 1.012 (0.986-1.037) | 0.087 |
| TBIL (μmol/L) | 1.032 (0.968-1.101) | 0.983 |
| DBIL (μmol/L) | 1.072 (0.885-1.299) | 0.984 |
| IBIL (μmol/L) | 1.061 (0.964-1.168) | 0.983 |
| **Kidney function test^c^** |  |  |
| Urea (mmol/L) | 0.792 (0.647-0.971) | 0.094 |
| UA (μmol/L) | 0.998 (0.994-1.002) | 0.598 |
| CR (μmol/L) | 0.995 (0.983-1.006) | 0.068 |
| CO2 (mmol/L) | 0.966 (0.842-1.109) | 0.463 |
| eGFR (mL/min) | 1.008 (1.000-1.016) | 0.005 |

^a^ NE: Neutrophils; LY: Lymphocytes; MO: Monocytes; HGB: Hemoglobin; PLT: Platelets; CRP: C-reactive protein.

^b^ ALT: Alanine aminotransferase; AST: Aspartate aminotransferase; TBIL: Total bilirubin; DBIL: Direct bilirubin; IBIL: Indirect bilirubin.

^c^ UA: Uric acid; CR: Creatinine; eGFR: estimated glomerular filtration rate.

**Table S4** Distribution and association of the height of fever in blood test, liver function test and kidney function test.

| **Characteristics** | **Afebrile** | **Low-grade fever** | **Moderate-grade fever** | **High-grade fever** | **H** | ***P* value** |
| --- | --- | --- | --- | --- | --- | --- |
| **Blood test^a^** |  |  |  |  |  |  |
| Leukocytes (10^9^/L) | 4.85 (3.93-6.18) | 5.56 (4.54-6.58) | 5.71 (4.96-6.94) | 5.52 (4.85-7.15) | 14.581 | 0.002 |
| NE (10^9^/L) | 3.47 (2.45-4.78) | 4.05 (3.00-5.04) | 4.40 (3.47-5.54) | 4.11 (3.46-6.00) | 18.629 | < 0.001 |
| LY (10^9^/L) | 0.97 (0.72-1.22) | 0.87 (0.64-1.21) | 0.86 (0.62-1.14) | 0.73 (0.63-1.07) | 5.227 | 0.153 |
| MO (10^9^/L) | 0.44 (0.35-0.54) | 0.46 (0.37-0.57) | 0.48 (0.37-0.59) | 0.45 (0.39-0.51) | 2.216 | 0.529 |
| HGB (g/L) | 134.00 (126.00-143.00) | 132.00 (123.00-143.00) | 135.00 (124.75-147.00) | 135.00 (124.25-144.75) | 3.627 | 0.305 |
| PLT (10^9^/L) | 206.00 (178.00-254.00) | 221.00 (185.00-264.75) | 212.00 (175.75-255.75) | 188.50 (163.75-246.00) | 4.728 | 0.193 |
| CRP (mg/L) | 9.63 (4.10-17.10) | 6.69 (3.34-15.98) | 9.86 (3.60-20.24) | 8.57 (3.49-18.65) | 1.330 | 0.722 |
| **Liver function test^b^** |  |  |  |  |  |  |
| ALT (U/L) | 20.40 (14.93-27.25) | 17.50 (13.25-27.90) | 19.70 (15.50-30.80) | 18.80 (15.33-30.55) | 3.865 | 0.276 |
| AST (U/L) | 24.30 (20.63-29.50) | 25.40 (20.20-33.25) | 25.20 (20.30-33.20) | 28.55 (23.73-33.40) | 4.870 | 0.182 |
| TBIL (μmol/L) | 8.10 (5.80-11.60) | 7.90 (5.95-10.60) | 8.85 (6.50-12.73) | 9.05 (6.73-14.00) | 4.832 | 0.185 |
| DBIL (μmol/L) | 3.60 (3.00-4.60) | 3.50 (2.80-4.50) | 3.80 (2.90-4.90) | 4.00 (3.50-5.58) | 5.032 | 0.169 |
| IBIL (μmol/L) | 4.70 (2.90-7.30) | 4.40 (2.85-6.25) | 5.00 (3.50-7.88) | 5.00 (3.45-8.28) | 5.421 | 0.143 |
| **Kidney function test^c^** |  |  |  |  |  |  |
| Urea (mmol/L) | 5.05 (3.92-6.40) | 4.24 (3.60-4.91) | 4.30 (3.60-5.10) | 4.45 (3.70-5.20) | 17.593 | 0.001 |
| UA (μmol/L) | 310.00 (247.75-352.25) | 287.00 (240.00-343.00) | 284.00 (247.00-348.00) | 262.00 (242.75-325.75) | 3.278 | 0.351 |
| CR (μmol/L) | 63.00 (53.00-78.00) | 54.00 (46.00-71.00) | 59.00 (45.00-75.00) | 52.50 (38.25-78.00) | 11.102 | 0.011 |
| CO2 (mmol/L) | 24.10 (23.10-25.20) | 23.80 (22.65-25.15) | 23.95 (22.20-25.50) | 23.60 (22.33-24.50) | 1.610 | 0.657 |
| eGFR (mL/min) | 100.42 (87.45-116.77) | 110.66 (99.19-139.29) | 110.76 (96.39-145.23) | 136.35 (97.08-172.91) | 17.637 | 0.001 |

^a^ NE: Neutrophils; LY: Lymphocytes; MO: Monocytes; HGB: Hemoglobin; PLT: Platelets; CRP: C-reactive protein.

^b^ ALT: Alanine aminotransferase; AST: Aspartate aminotransferase; TBIL: Total bilirubin; DBIL: Direct bilirubin; IBIL: Indirect bilirubin.

^c^ UA: Uric acid; CR: Creatinine; eGFR: estimated glomerular filtration rate.

**Table S5** Multivariate logistic regression analysis of blood test, liver function test and kidney function test across the fever severity groups.

| **Characteristics** | **Multivariate** | |
| --- | --- | --- |
|  | **OR (95%*CI*)** | ***P* value** |
| **Blood test^a^** |  |  |
| Leukocytes (10^9^/L) | 1.112 (0.000-10341.850) | 0.982 |
| NE (10^9^/L) | 1.420 (0.000-14190.068) | 0.941 |
| LY (10^9^/L) | 0.982 (0.000-18749.913) | 0.988 |
| MO (10^9^/L) | 0.039 (0.000-757.716) | 0.521 |
| HGB (g/L) | 0.993 (0.971-1.015) | 0.511 |
| PLT (10^9^/L) | 0.995 (0.989-1.001) | 0.100 |
| CRP (mg/L) | 1.019 (0.995-1.042) | 0.116 |
| **Liver function test^b^** |  |  |
| ALT (U/L) | 0.978 (0.944-1.014) | 0.225 |
| AST (U/L) | 1.042 (0.995-1.092) | 0.083 |
| TBIL (μmol/L) | 0.007 (0.000) | 0.999 |
| DBIL (μmol/L) | 157.438 (0.000) | 0.999 |
| IBIL (μmol/L) | 134.829 (0.000) | 0.999 |
| **Kidney function test^c^** |  |  |
| Urea (mmol/L) | 0.814 (0.629-1.052) | 0.115 |
| UA (μmol/L) | 1.001 (0.997-1.006) | 0.545 |
| CR (μmol/L) | 1.041 (1.014-1.069) | 0.003 |
| CO2 (mmol/L) | 0.952 (0.828-1.093) | 0.485 |
| eGFR (mL/min) | 1.043 (1.025-1.062) | < 0.001 |

^a^ NE: Neutrophils; LY: Lymphocytes; MO: Monocytes; HGB: Hemoglobin; PLT: Platelets; CRP: C-reactive protein.

^b^ ALT: Alanine aminotransferase; AST: Aspartate aminotransferase; TBIL: Total bilirubin; DBIL: Direct bilirubin; IBIL: Indirect bilirubin.

^c^ UA: Uric acid; CR: Creatinine; eGFR: estimated glomerular filtration rate.

**Table S6**  Multivariate logistic regression analysis of demographic characteristics for prolonged Chikungunya fever.

| **Characteristics** | **Multivariate** | |
| --- | --- | --- |
|  | **OR (95%CI)** | ***P* value** |
| Age | 1.022 (1.012-1.030) | < 0.001 |
| Gender | 1.012 (0.672-1.527) | 0.954 |
| Hypertension | 1.090 (0.510-2.364) | 0.825 |
| Hyperlipidemia | 0.455 (0.102-1.828) | 0.267 |
| Diabetes | 1.044 (0.354-3.157) | 0.938 |
| Chronic gastritis/Peptic ulcer | 1.209 (0.319-4.594) | 0.774 |
| Duration of fever | 1.199 (0.942-1.527) | 0.141 |
| Clinical symptoms | 0.907 (0.652-1.262) | 0.563 |

**Table S7**  Multivariate logistic regression analysis of blood test, liver function test and kidney function test for prolonged Chikungunya fever.

| **Characteristics** | **Multivariate** | |
| --- | --- | --- |
|  | **OR (95%CI)** | ***P* value** |
| **Blood test^a^** |  |  |
| Leukocytes (10^9^/L) | 0.956 (0.035-25.863) | 0.979 |
| NE (10^9^/L) | 0.881 (0.028-28.079) | 0.944 |
| LY (10^9^/L) | 2.060 (0.034-125.909) | 0.721 |
| MO (10^9^/L) | 1.691 (0.107-26.797) | 0.384 |
| HGB (g/L) | 0.992 (0.978-1.006) | 0.279 |
| PLT (10^9^/L) | 0.992 (0.987-0.996) | < 0.001 |
| CRP (mg/L) | 1.007 (0.991-1.024) | 0.374 |
| **Liver function test^b^** |  |  |
| ALT (U/L) | 0.998 (0.986-1.010) | 0.708 |
| AST (U/L) | 1.008 (0.992-1.025) | 0.300 |
| TBIL (μmol/L) | 0.011 (0.001-1.516) | 0.190 |
| DBIL (μmol/L) | 1.215 (0.001-2.287) | 0.225 |
| IBIL (μmol/L) | 1.321 (0.001-2.484) | 0.147 |
| **Kidney function test^c^** |  |  |
| Urea (mmol/L) | 1.013 (0.852-1.205) | 0.880 |
| UA (μmol/L) | 0.998 (0.995-1.001) | 0.180 |
| CR (μmol/L) | 0.995 (0.977-1.012) | 0.543 |
| CO2 (mmol/L) | 1.020 (0.917-1.135) | 0.709 |
| eGFR (mL/min) | 0.990 (0.987-0.998) | 0.046 |

^a^ NE: Neutrophils; LY: Lymphocytes; MO: Monocytes; HGB: Hemoglobin; PLT: Platelets; CRP: C-reactive protein.

^b^ ALT: Alanine aminotransferase; AST: Aspartate aminotransferase; TBIL: Total bilirubin; DBIL: Direct bilirubin; IBIL: Indirect bilirubin.

^c^ UA: Uric acid; CR: Creatinine; eGFR: estimated glomerular filtration rate.
